# A novel mentorship programme for residents integrating academic development, clinical teaching and graduate medical education assessment

**DOI:** 10.1007/s40037-015-0236-2

**Published:** 2016-01-26

**Authors:** Kriti Bhatia, James Kimo Takayesu, Eric S. Nadel

**Affiliations:** Department of Emergency Medicine, Brigham & Women’s Hospital, Harvard Medical School, 75 Francis Street, 02115 Boston, MA USA; Department of Emergency Medicine, Massachusetts General Hospital, Boston, USA; Department of Emergency Medicine, Brigham & Women’s Hospital/Massachusetts General Hospital, Boston, USA

**Keywords:** Mentorship, Academic development, Assessment, Direct observation

## Abstract

**Introduction:**

Mentorship fosters career development and growth. During residency training, mentorship should support clinical development along with intellectual and academic interests. Reported resident mentoring programmes do not typically include clinical components. We designed a programme that combines academic development with clinical feedback and assessment in a four-year emergency medicine residency programme.

**Methods:**

Incoming interns were assigned an advisor. At the conclusion of the intern year, residents actively participated in selecting a mentor for the duration of residency. The programme consisted of quarterly meetings, direct clinical observation and specific competency assessment, assistance with lecture preparation, real-time feedback on presentations, simulation coaching sessions, and discussions related to career development. Faculty participation was recognized as a valuable component of the annual review process. Residents were surveyed about the overall programme and individual components.

**Results:**

Over 88 % of the respondents said that the programme was valuable and should be continued. Senior residents most valued the quarterly meetings and presentation help and feedback. Junior residents strongly valued the clinical observation and simulation sessions.

**Conclusions:**

A comprehensive mentorship programme integrating clinical, professional and academic development provides residents individualized feedback and coaching and is valued by trainees. Individualized assessment of clinical competencies can be conducted through such a programme.

## Introduction

Mentorship is a well-developed practice in fields such as business and the military [1]. Mentorship in medicine has become increasingly important as a catalyst for academic development for faculty and residents seeking careers in academic medicine [2, 3]. Mentoring specifically for residents deserves consideration since training is a period of rapid growth requiring time-sensitive decisions about career and practice choices in addition to achieving competence in patient care skills [4, 5]. Although numerous mentorship programmes targeting residents have been developed, reported programmes do not typically include clinical mentoring components despite evidence that trainees’ clinical skills need improvement.

In 2001, the United States’ Accreditation Council of Graduate Medical Education (ACGME) introduced six core competencies to standardize the evaluation of resident performance [6]. Their implementation has required a significant time commitment from residency programmes for direct observational assessment of clinical skills [6]. Successful academic careers require a variety of non-clinical skills including writing, presentation preparation, lecturing, research methodology, and innovation. Identification and development of an area of academic interest is an important prerequisite for an academic position [7]. One study demonstrated emergency medicine residents’ perceptions of their training in such skills to only be fair [8]. Engaging faculty mentors to perform direct observational assessments and to aid in academic development can provide residents with valuable, individualized feedback that might not otherwise happen if left to the residency administrative structure alone.

We designed a mentorship programme, Mentorship for Clinical and Academic Development, integrating development of essential academic skills and the ACGME core competencies to enhance the resident learning experience.

## Methods

### Setting and participants

The Harvard Affiliated Emergency Medicine Residency is a 60 resident, four-year programme. Residents staff two tertiary-care facilities and rotate at affiliated paediatric and community institutions. The mentoring pool consists of approximately 60 physicians.

### The programme

Incoming interns are assigned a first-year advisor, typically a junior faculty member given their proximity to the training experience. Additionally, involving junior faculty provides them experience early in their careers to foster a ‘culture of mentorship’ within the faculty pool. These pairings were assigned by the associate programme director in a double-blinded fashion accounting for resident preferences and faculty interests. The required components of the programme include:

- Quarterly development meetings—to discuss the transition to internship and identify potential area(s) of academic interest. Personal growth and development are also addressed.
- Simulation session—a 1:1 session for which faculty may bring a case or choose from an available case bank. The goal of this session is twofold: to provide residents with valuable direct observation and individualized, immediate feedback as well as to create an opportunity for faculty assessment and input for residents’ portfolios to be used for promotion decisions.
- Direct clinical observation session—Interns are observed for at least three complete patient evaluations during a time when advisors have no clinical duties. Verbal feedback is provided. The Standardized Direct Observation Tool, an assessment tool developed by the Council of Emergency Medicine Residency Directors for clinical skills and core competencies evaluation, is completed.
- Presentations—Advisors are available to help with the preparation and review of all presentations prior to delivery. Faculty attend presentations barring unavoidable conflicts and provide verbal feedback.

At the end of first year, interns provide a list of three possible mentors for the remainder of training. Based on overall requests and faculty availability, they are assigned one of these mentors. The required components of the programme are the same as those for the intern year although the quarterly meetings are more focused on specific research or scholarly projects and issues related to career development. Mentors are asked to complete forms after each encounter, summarizing the discussion points and assessment if applicable.

During the fourth postgraduate year, mentors serve as resources for the job search process. In addition to these required activities, faculty mentors are involved if there is a particular concern that arises, such as remediation for in-training examination scores, professionalism issues, or cognitive or procedural deficits, to ensure the resident has support beyond the residency administration.

### Survey

Four years after the initiation of the Mentorship for Clinical and Academic Development programme, a survey was administered to current residents and recent residency graduates who had participated in the programme. Participants were asked about the overall quality of the programme as well as about each component. A survey was developed by the residency administration. Questions were reviewed by a focus group of five faculty and three chief residents for content and wording prior to distribution. It was declared exempt by the Institutional Review Board.

## Results

The overall response rate was 86.67 % (*n* = 60). Of the respondents, 88.1 % thought that the programme was valuable and should be continued. The most positively viewed components were the quarterly meetings, assistance with presentation preparation, and mentor attendance at presentations. Junior residents valued the simulation session strongly. A total of 43.9 % of the respondents thought that shared academic interest was most important to a successful mentor-mentee match while 43.9 % thought that a comfortable personal dynamic was most important; 12.2 % had no preference (Table 1).

**Table 1:** Resident-reported comfort level with addressing specific topics with mentor

Topic	Score^a^
Research or career-development	6/6
Clinical issues or performance	5.2/6
Personal study or reading plan development	4.1/6
Personal issues (financial, health, interpersonal relationships)	3.1/6
Talks or presentations	6/6
Job prospects	5.8/6

^a^Scores are based on a scale of 1–6 with 6 being the most comfortable.

## Discussion

Shortly after its inception, our residency programme introduced a mentorship programme through which residents were paired with a faculty mentor based solely on faculty availability. While this programme occasionally resulted in a fruitful relationship, these mentoring pairs usually lived on paper but not in practice. We implemented a novel mentorship programme that integrates aspects of existing models of mentorship in medicine, resident needs for academic development, and the core competency assessment and evaluation requirements of graduate medical education.

The Mentorship for Clinical and Academic Development programme has been well received because it is multi-faceted, incorporating a variety of components focusing on the individual resident’s longitudinal growth and development. Existing literature refutes the belief that mentors should provide all the answers in all domains for mentees [9]. While recognizing this, we designed the Mentorship for Clinical and Academic Development programme as a means to assign responsible faculty to enhance resident development in areas that all practising emergency physicians have developed through experience and teaching. Mentors are encouraged to refer residents to additional faculty to broaden their mentoring network and connection with experts for academic interest development and personal growth.

An individual’s active participation in mentor selection can yield better outcomes, as evidenced by the even split among residents feeling interpersonal dynamics and academic interests are the most essential in a successful mentor selection process [10]. Since spontaneous mentoring pairs do not always develop, the programme provides the structure and support to ensure that effective mentoring pairs are created. Providing specific mentoring requirements gives structure to the relationship that fosters outcome-oriented meetings and accountability for each of the specific learning activities.

Competing time demands for the faculty, the lack of acknowledgment of time devoted to mentorship, and the pressures for faculty scholarship perpetually challenge mentoring relationships [2, 9, 11]. The residency administration submits completion data about the various programme requirements to the chairmen for use in annual faculty performance review. In our institutions, mentorship is a recognized and rewarded position, making it a factor in the assessment of a faculty member’s contributions and financial remuneration. This helps make explicit the goal of creating a culture of mentorship in the residency and faculty communities.

Having faculty mentors directly observe residents provides valuable real-time feedback for residents. Direct observation allows assessment of residents’ patient care and professional skills that can often be precluded by the busy clinical environment [12]. Such individualized coaching may also enhance personal development by imparting more self-confidence [13].

There were a number of limitations to our programme and subsequent study. The programme was implemented with a relatively small group of residents from one speciality. Broader application in additional residencies across specialities would identify additional challenges and successful components of the programme that could guide speciality-specific integration recommendations. We did not use a standardized programme evaluation. Our data were collected retrospectively through a survey and did not include direct comparison between prior mentorship programmes and our Mentorship for Clinical and Academic Development programme. In addition, we did not survey the mentors to identify their perceptions of programme strengths and shortcomings.

The literature supports formal training of mentors to enhance the mentoring relationship. To date, our programme has not included formal education of mentors; we plan to develop such a module for future mentors. Given the already numerous demands on faculty, mandating a curriculum and gathering all mentors together can be difficult. Therefore we anticipate offering an on-line educational programme that can be completed independently.

Despite competing time demands, faculty participation in the Mentorship for Clinical and Academic Development programme has remained robust since its inception. Though a formal survey has not been conducted, faculty anecdotally cite personal satisfaction by investing in a mentee as their primary motivation. Acknowledgement and financial support of this role in the annual review process helps ensure sustainability.

Though our programme was developed for and implemented in an emergency medicine residency, the principles and components are easily applicable to any speciality with minor programme-specific modification.

## Conclusions

A multi-faceted mentorship programme should integrate elements of clinical development, academic growth, and direct observational assessments. We have created and implemented such a programme for emergency medicine residents in a four-year academic residency. Although requiring a significant time commitment from faculty, rewarding participation through the annual review process can enhance participation and accountability. Residents receive valuable individualized teaching and feedback in areas that are critical for career development. By including direct observational components, this programme can also support the formative assessments of clinical performance to meet ACGME training requirements and good educational practice.
